# Development and preliminary evaluation of the Comprehensive Health Self-Assessment Questionnaire (CHSAQ) for individuals in the Chinese People Liberation Army

**DOI:** 10.1186/s12889-024-18085-w

**Published:** 2024-02-26

**Authors:** Tao Wang, Han Tang, Xinrui Li, Lin Wu, Ning Li, Wei Zhang, Qiongjie Shao, Min Cai, Lei Shang

**Affiliations:** 1 Department of Health Statistics, School of Public Health, the Fourth Military Medical University, 710032 Xi’an, Shaanxi China; 2 The Medical Department, Xijing Hospital, the Fourth Military Medical University, Xi’an, Shaanxi China; 3 Department of Military Medical Psychology, the Fourth Military Medical University, Xi’an, Shaanxi China; 4 Department of Psychiatry, Xijing Hospital, the Fourth Military Medical University, 710032 Xi’an, Shaanxi China; 5 Department of Thoracic Surgery, Tangdu Hospital, the Fourth Military Medical University, Xi’an, Shaanxi China

**Keywords:** Self-report questionnaire, Comprehensive health, PLA, Reliability, Validity

## Abstract

**Objective:**

There is currently no widely accepted multidimensional health assessment questionnaire for individuals in the Chinese People Liberation Army (PLA). This study developed a multidimensional health survey questionnaire (Comprehensive Health Self-Assessment Questionnaire, CHSAQ) suitable for personnel in the PLA and conducted a preliminary examination of its reliability, validity, and discriminative ability.

**Methods:**

After 183 items from 32 dimensions were selected to form the initial version of the CHSAQ, three groups of soldiers were selected from May 2022 to April 2023 and completed three survey rounds (with 183, 131, and 55 valid items). The items were screened based on classic test theory. After screening, the final questionnaire entries were formed, the structure of the questionnaire was explored through exploratory factor analysis and confirmatory factor analysis, and its reliability, structural validity, and discriminative ability were evaluated.

**Results:**

The final questionnaire consisted of 8 dimensions and 55 items on job satisfaction, anxiety and depression, daily activities, physical function, the otolaryngology system, the integumentary system, sleep disorders, and the visual system. The total cumulative variance contribution rate was 64.648% according to exploratory factor analysis. According to the confirmatory factor analysis, the normed fit index (NFI) was 0.880, and the comparison fit index (CFI) was 0.893 (close to 0.90). The Cronbach’s α coefficient of the total questionnaire was 0.970, the split half reliability coefficient was 0.937, and the retest reliability coefficient was 0.902. The results are presented as different pairwise comparisons.

**Conclusion:**

Our study developed a self-report questionnaire for evaluating the comprehensive health status of personnel in the PLA in accordance with the standard procedure for questionnaire development. Our findings also showed that the CHSAQ for individuals in the PLA has good reliability and structural validity.

## Background

As a special occupational group, military personnel have higher health standards than ordinary people [1–3]. The level of health is crucial for military workers’ competitiveness at work, ability to handle interpersonal relationships and ability to gain support from individuals outside the military [4, 5]. In recent decades, people’s understanding of health has shifted from the biomedical model to the biopsychosocial medical model [6, 7]. Therefore, military health managers need to assess the health of military workers not only on the basis of traditional training consultation, trauma treatment, disease diagnosis and treatment, and psychological support but also in terms of social and mental health [8–11].

Many studies have attempted to conduct military health surveys through questionnaires. A questionnaire can be used to evaluate research subjects from different perspectives through a series of related questions [12–14]. For example, self-report questionnaires, such as the Medical Outcomes Study 36-Item Short Form Health Survey (SF-36 C), which was developed by the Boston Institute of Health in the United States (U.S.), have been adopted by many researchers for their ease of understanding and ability to reflect individuals’ evaluations and expectations of their health status [15–17]. On this basis, some developed countries have developed comprehensive military health assessment questionnaires, such as the Global Assessment Tool (GAT), developed in the U.S [18, 19].. This health survey questionnaire for military personnel takes approximately 15 min to complete and includes a total of 105 items and four dimensions, namely, emotional health, family health, social health, and mental health. This questionnaire has been developed by the U.S. government and was improved upon in subsequent research by adding age and sex stratification, as well as items regarding personality strength and psychological resilience [20]. According to the research of Loryana L. Vie and Lawrence M. Scheier, the improved GAT was found to have good reliability and validity and to be a reliable and scientific multidimensional health assessment tool [21]. Other countries, such as Canada, have also developed their own health questionnaire tools for soldiers or veterans [22].

Several studies have investigated health literacy and its influencing factors in individuals in the People Liberation Army (PLA) [23]. However, to our knowledge, there is no health questionnaire that meets the characteristics of the Chinese PLA. Therefore, this study was designed to develop a comprehensive health self-assessment questionnaire (CHSAQ) that conforms to the characteristics of individuals on the Chinese PLA and to conduct preliminary tests on its reliability, validity, and discrimination ability. The hypothesis of the current study was that we could obtain a self-reported CHSAQ with good reliability and validity in accordance with the standard procedure for questionnaire development.

## Materials and methods

### Participants

**Fig. 1:**
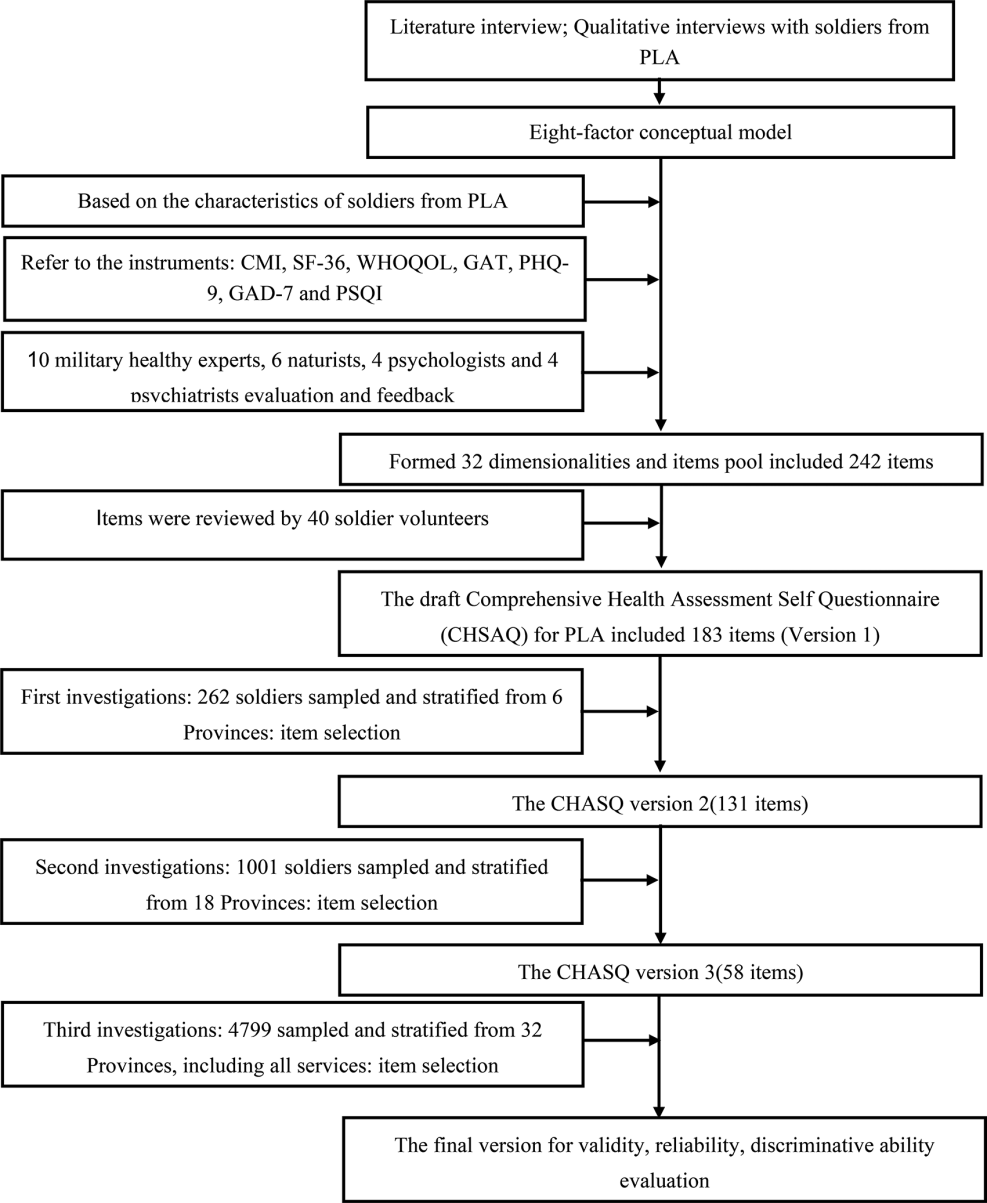
The flow chart of the whole process of development and preliminary evaluation of Comprehensive Health Self-Assessment Questionnaire (CHSAQ). CMI, Cornell Medical Index; SF-36, 36-item Short-Form; WHOQOL, The World Health Organization Quality of Life; GAT, Global Assessment Tool; PHQ-9, Patient Health Questionnaire-9; GAD-7, Generalized Anxiety Disorder 7; and PSQI, Pittsburgh sleep quality index

The present study was conducted in three rounds from May 2022 to April 2023. To obtain a representative sample of soldiers with varying weight statuses, the researchers included volunteers from various areas of China. All participants were enrolled according to the following criteria: (a) aged ≥ 18 years, (b) served in the army, and (c) provided informed consent. The exclusion criteria were as follows: (a) soldiers with severe disease and (b) individuals who did not provide informed consent. To provide a clear overview of the procedure in this study, a flowchart was constructed, as shown in Fig. 1.

Prior to the formal investigation, a clear explanation of the aim of this study and its procedure was distributed to each participant to help them fully understand the purpose and significance of this study, as well as the meaning of each item, the instructions for completing the questionnaires, and a description of the examination methods. All recruited participants provided written informed consent before the collection of any information, and all methods were performed in accordance with the relevant guidelines and regulations. The present study was approved by the Research Ethics Committee of the Fourth Military Medical University (code number: K202305-41).

### Conceptual model selection and development of the draft version of the CHSAQ

Through two rounds of qualitative interviews, a conceptual model was developed to summarize the CHSAQ in a comprehensive way. The first round of qualitative interviews collected information on comprehensive health status to the greatest extent possible through in-depth individual interviews with 40 participants. After summarization of the collected information, an interview outline addressing health instruments was created for use in a second round of qualitative interviews. The second-round qualitative interview consisted of a focus group discussion among the 40 participants and addressed the universality of the items on the general health instrument outline. The results from the focus group were subsequently used to establish the conceptual model. The model included the following 32 dimensions: 16 physical fitness dimensions (stomatology, ophthalmology, otolaryngology, respiratory system, circulation system, digestive system, orthopaedics, integumentary system, nervous system, urogenital system, daily activities, physical activities, pain, nutrition and metabolism, tobacco use, and military training); 15 psychological and psychiatric health dimensions (depression, anxiety, sleep, mood stability, obsessive-compulsive and psychotic behaviour, fatigue, job satisfaction and cognitive function, environment adaptability, coping styles, personality, social interaction, life satisfaction, family relationships, and social support); and 1 overall assessment dimension.

In accordance with the conceptual model framework and previously reported instruments, including the Cornell Medical Index (CMI) [24], 36-item Short Form Health Survey (SF-36), World Health Organization Quality of Life (WHOQOL) questionnaire [25], GAT, Patient Health Questionnaire-9 (PHQ-9) [26], Generalized Anxiety Disorder-7 (GAD-7) [27] and Pittsburgh Sleep Quality Index (PSQI) [28], 219 items were selected for the item pool. We referred to domestic and foreign questionnaires about the health status of adults and soldiers, mainly referring to the purpose and content of their evaluations; then, we constructed an item pool based on the training characteristics of Chinese soldiers. After referring to other questionnaires, to ensure the localization and specificity of the item content for soldiers in China, we conducted two rounds of focus group interviews. In the first round of interviews, 20 soldiers who underwent physical examination were selected by the convenience sampling method. A second interview was conducted with 10 military health specialists, 6 nutrition specialists, 4 psychologists, and 4 psychiatrists. According to the characteristics of Chinese soldiers, the participants’ opinions and suggestions were summarized; 23 items related to Chinese military training and the physical function of soldiers were supplemented; and the content validity, relevance, clarity and ambiguity of the items were also evaluated. A written report was provided by each reviewer. In this manner, an item pool including 242 items was established.

This item pool was subsequently reviewed by 40 soldiers who visited Xijing Hospital for annual medical examination. Each item was evaluated in terms of its importance (illustrated by the correlation with comprehensive health status) and frequency. Each item was scored based on a Likert-type questionnaire (with 12 questions) ranging from 1 (not very important or rare) to 5 (very important or very frequent); thus, each item had a total possible score of 60 for importance and frequency. An item’s importance or frequency increased as its mean score increased. Items that met the following criteria were removed: a frequency × importance score < 50th percentile plus an importance score < 50th percentile and a frequency-× importance score < 50th percentile plus a frequency score < 50th percentile. After this review, 59 items were deleted because the respondents believed that they were either frequent or important. Ultimately, a draft version of the CHSAQ that included 183 items was created.

### Scoring methods

Each item of the questionnaire measures the frequency/importance of each status in relation to physical/psychological/psychiatric health conditions over the previous 4 weeks. Five answer options were developed for different items through response dimension analysis: “always”, “often”, “sometimes”, “never” and “rarely”. The response options are assigned values of 1, 2, 3, 4, or 5, and the total possible score ranges from 55 to 275 points. The higher the total score is, the better a soldier’s health status.

The mean score is expressed as the sum of the items divided by the number of.

items answered in each dimension, and the sum of the scores in each dimension was used to determine the total score.

### Investigation methods

In this study, all of the three investigations were conducted by three investigators who were trained before the start of each inquiry. During the study, the investigators explained the purpose and the whole process of the investigation and the significance of the questionnaires to participants who agreed to participate. Second, the questionnaires were distributed to the participants individually. Based on their health status over the past 4 weeks, the participants completed the questionnaires and returned them to the investigators.

All the questionnaires were carefully double-checked by the investigators, and telephone interviews were conducted to collect missing information from participants with incomplete questionnaires. To ensure data accuracy, the double-entry method was used, and logic checks for errors were confirmed. All the data were collected anonymously.

#### Development of the trial questionnaire (for the first investigation)

For the first investigation, a convenience sample of 317 soldiers from 6 provinces was included, and predefined investigation methods and inclusion and exclusion criteria were applied. All participants independently completed the first draft of the CHSAQ and returned it to the investigators. This sample was used for item analysis and construction of the draft version of the CHSAQ.

#### Development of the final questionnaire (for the second investigation)

In Sample 2, 1001 informants from 18 provinces (including the Army, Navy, and Air Force) participated in this investigation. The methods and inclusion and exclusion criteria were the same as those for Sample 1. All participants completed the draft version of the questionnaire and returned it to the investigators. This sample was used for item analysis of the draft questionnaire and for construction of the final version of the CHSAQ.

#### Evaluating the final questionnaire (for the third investigation)

Sample 3 consisted of 4799 participants from 32 provinces recruited via convenience sampling from March 2023 to May 2023; all armed services in 5 military regions of the PLA were sampled using the same methods and inclusion and exclusion criteria as in Samples 1 and 2. Each participant independently completed the final version of the CHSAQ and returned it to the investigators. This sample was used for the final determination of the questionnaire’s dimensions and the analysis of its reliability, validity, and discriminative ability. A subset of 750 (15% of all participants in this round) survey informants from this sample was randomly selected to complete the CHSAQ again after 2 weeks.

### Data analysis

All completed questions that were included for the determination of validation were entered into a database built with SPSS software (SPSS 22.0). First, items were removed when more than 15% of the survey participants received the highest or lowest score, which indicates floor effects or ceiling effects. Second, reverse-scored items were converted according to the following rules (5 = 1, 2 = 4, 4 = 2 and 1 = 5). The item selection methods were as follows [29–31]. (1) The critical ratio analysis method: The total score of the scale was obtained by adding all item scores and ranking them in descending order. According to 27% of the total number, the total score was calculated after the top and bottom 27% of the population were divided into two groups. Afterwards, using an independent sample *t* test to analyse the difference in each item between the two groups, those with a final *P* value > 0.05 were removed. (2) For the discrete trend method, items with a standard deviation of less than 0.85 (from the score) were removed. (3) For the correlation coefficient method, items with a correlation coefficient between each item and the total score of the questionnaire less than 0.4 or with a *P* value > 0.05 were removed. (4) For the exploratory factor analysis (EFA) method, items with a factor loading < 0.4 or multiple-factor loading > 0.4 were removed. (5) For the Cronbach’s alpha coefficient method, items with a corrected item-total correlation (CITC) < 0.3 were removed. Items that met ≥ 4 of the above criteria were retained; otherwise, they were removed.

### Reliability analysis

Reliability was evaluated by calculating the Cronbach’s α coefficient, test-retest reliability coefficient, and split-half reliability coefficient. When the α coefficient of the total questionnaire was greater than or equal to 0.70 and the α coefficient of the dimension was greater than or equal to 0.60, the result was considered satisfactory [29].

### Validity analysis

The evaluation of the content validity of the questionnaire was confirmed by the expert consultation method. In brief, a two-step strategy of model building was used to examine the validity of the CHSAQ. First, half of the sample was randomly selected for EFA to extract the factors. Second, the other half of the sample was used to confirm the factor structure of the questionnaires via confirmatory factor analysis (CFA) [30].

To determine if the factor model fit the data from each sample well, the investigators focused on seven fit indices according to previous reports: the standardized root mean squared residual (*SRMR*), the *χ*^2^/*df*, the nonnormed fit index (*NNFI*), the comparative fit index (*CFI*), the goodness-of-fit index (*GFI*), the adjusted goodness-of-fit index (*AGFI*), and the root mean square error of approximation (*RMSEA*) [31]. Generally, a relatively good model-data fit was defined as follows: *an SRMR* smaller than 0.08, a *CFI* and an *AGFI* larger than 0.90, and *an NNFI* and a *GFI* larger than 0.95. The *χ*^2^/*df* ratio analyses the fit of a model by comparing the obtained sample correlation matrix with the correlation matrix estimated under the model. A lower *χ*^2^/*df ratio* indicates a better fit, as reflected by the small discrepancy between the structure of the observed data and the hypothesized model. Because the *χ*^2^/*df* ratio is extremely sensitive to sample size, e.g., a value less than 5 indicates increasingly good fit, additional fit indices were considered. The *RMSEA* indicates how close the model fit approximates a reasonably fitted model, and a value < 0.05 indicates good model fit.

### Statistical analysis of discrimination

The characteristics of the quantitative data are expressed as the mean ± standard deviation (SD), and the characteristics of the qualitative data are expressed as the number (*n*) and proportion (%). In the present study, a two-sample *t* test or one-way analysis of variance was used to compare scores on different dimensions across individuals in the PLA with different sexes, ages, educational levels, body mass indices (BMIs), years of service and work environments. The normality of all the data in this study was evaluated with descriptive evidence from a one-sample Kolmogorov‒Smirnov test. A two-tailed *P* value < 0.05 was considered to indicate statistical significance.

## Results

### Demographic characteristics of the participants

All demographic characteristics of the three samples are shown in Table 1. In the first investigation, a total of 280 soldiers were enrolled by stratified sampling methods, and 93.6% (262/280) of the questionnaires were valid. In the second round of evaluation, 1200 soldiers were enrolled, and 91% (1001/1100) of the valid questionnaires were completed. The final questionnaire consists of 8 dimensions and 55 items related to job satisfaction (14 items for examining the satisfaction of military individuals with their work), anxiety and depression (12 items for the assessment of anxiety and depression levels of military participants), daily activities (10 items were used to evaluate the functional level of participants during daily activities), physical function (3 items for analysing the physical fitness of explosive power, flexibility and reactivity when exercising or training military participants), the otolaryngology system (4 items for examining the symptoms of runny, nose congestion, sneezing, and severe cold of military participants), the integumentary system (4 items for evaluating the symptoms of rash, pruritus, allergy and haemorrhagic spots of military participants), sleep disorders (4 items for examining sleep disorder symptoms regarding dysphylaxia, dreaminess, insomnia, and night-timeurination in participants), and the visual system (4 items were selected for evaluating symptoms in the visual system, including sore eyes, blurring of vision, red eyes, blinks and weeping eyes). In the last round, 5000 soldiers were enrolled, and 95.9% (4799/5000) of the valid questionnaires were completed.

**Table 1:** Demographic information for soldiers participated the evaluation of the Comprehensive Health Assessment Self Questionnaire (CHASQ) of PLA, 2022–2023

Group	Sample 1 (*n* = 262)		Sample 2 (*n* = 1001)		Sample 3 (*n* = 4799)	
	n	%	n	%	n	%
**Gender**						
Male	251	95.8	839	83.8	4361	90.9
Female	11	4.2	162	16.2	438	9.1
**Age (years)**						
18 ~ 30	209	79.8	629	62.8	3751	78.2
31 ~ 40	40	15.3	307	30.7	810	16.9
41~	13	4.9	65	6.5	238	4.9
**Nationality**						
Han nationality	249	95.0	975	97.4	4389	91.5
Minority nationality	13	5.0	26	2.6	410	8.5
**Urban or rural sources**						
Rural	167	63.7	627	62.6	3612	75.3
Urban	95	36.3	374	37.4	1187	24.7
**Marital status**						
Mateless	193	73.7	536	53.5	3401	70.9
Spouse	69	26.3	465	46.5	1398	29.1
**Quantity of children**						
0	208	79.4	637	63.6	3752	78.2
1	40	15.3	270	27.0	729	15.2
2	10	3.8	59	5.9	287	6.0
≥3	4	1.5	35	3.5	31	0.6
**Years of service**						
0 ~ 10	201	76.7	633	63.2	3821	79.6
11 ~ 20	39	14.9	273	27.3	751	15.6
21~	22	8.4	95	9.5	227	4.8
**Nature of work**						
Administrative	74	28.2	423	42.3	2161	45.0
Technical	188	71.8	578	57.7	2638	55.0
**Title**						
Junior and below	243	92.7	795	79.4	4113	85.7
Intermediate	15	5.7	163	16.3	516	10.8
Deputy Senior or above	4	1.6	43	4.3	170	3.5
**Education**						
Below bachelor degree	182	69.5	381	38.1	2947	61.4
Bachelor degree	65	24.8	410	41.0	1499	31.2
Master degree or above	15	5.7	210	21.0	353	7.4
**Grade of BMI**						
Underweight (<18.5 kg/m^2^)	5	1.9	29	2.9	152	3.2
Normal (18.5 ~ 23.9 kg/m^2^)	159	60.7	612	61.1	3169	66.0
Overweight (24.0 ~ 27.9 kg/m^2^)	90	34.3	320	32.0	1359	28.3
Obesity (≥28.0 kg/m^2^)	8	3.1	40	4.0	119	2.5
**Smoking situation**						
Never	155	59.2	627	62.6	2685	55.9
1–5 per day	64	24.4	136	13.6	1041	21.7
6–10 per day	29	11.0	124	12.4	569	11.9
11–20 per day	13	5.0	101	10.1	443	9.2
21 or above per day	1	0.4	13	1.3	61	1.3
**Drinking situation**						
Never	161	61.4	357	35.7	3232	67.3
Occasionally	99	37.8	427	42.7	1544	32.2
Often	2	0.8	217	21.6	23	0.5
**Exercise situation**						
Never	7	2.7	33	3.3	49	1.0
Occasionally	56	21.3	323	32.3	1390	29.0
Often	199	76.0	645	64.4	3360	70.0
**Allergic foods**						
No	246	93.9	921	92.0	4598	95.8
Yes	16	6.1	80	8.0	201	4.2
**Allergic drugs**						
No	243	92.7	929	92.8	4588	95.6
Yes	19	7.3	72	7.2	211	4.4
**Work area**						
Plain	207	79.0	880	87.9	2924	60.9
Mountainous region	22	8.4	28	2.8	912	19.0
Hill	21	8.0	19	1.9	380	7.9
Basin	4	1.5	13	1.3	146	3.0
Desert	3	1.1	33	3.3	127	2.6
Plateau	5	2.0	28	2.8	310	6.6
**Geographical distribution**						
East	2	0.8	32	3.2	1233	25.7
South	5	1.9	20	2.0	400	8.3
West	78	29.8	764	76.3	1271	26.5
North	174	66.4	160	16.0	892	18.6
Middle	3	1.1	25	2.5	1003	20.9
**Regional climate**						
Warm region	218	83.2	998	99.7	3150	65.6
Cold region	12	4.6	2	0.2	1296	27.0
Hot region	29	11.1	1	0.1	291	6.1
Others	3	1.1	0	0	62	1.3
**Work environment**						
Normal environment (indoor, plain, et al.)	83	31.7	660	66.0	3651	76.1
Extreme weather (high temperature, high humidity, extreme cold)	87	33.2	124	12.4	522	10.9
Confined space (compartment, cave, et al.)	44	16.8	131	13.0	177	3.7
Others (aviation, sea diving, frontier defense, island, et al.)	48	18.3	86	8.6	449	9.3
**Eating habits**						
Health (regular diet, reasonable collocation, et al.)	126	48.1	423	42.3	4723	98.4
Unhealthy (irregular diet, eat and drink too much, et al.)	136	51.9	578	57.7	76	1.6
**Harmful factors**						
No	137	52.3	571	57.0	4259	88.7
Yes (noise, electromagnetic radiation, toxic and harmful substances, et al.)	125	47.7	430	43.0	540	11.3

### Exploratory factor analysis

With the use of the “random case selection” function in the SPSS system, 2396 participants were selected from 4799 participants, and the 55 questionnaire items were analysed via EFA. The Kaiser–Meyer–Olkin (KMO) test value was 0.980, the Bartlett sphericity test value was 91910.423, and the accompanying probability was far less than the significance level of 0.05, which met the conditions of factor analysis. The results of the factor analysis showed that 8 factors needed to be extracted. The factor load matrix after orthogonal rotation of the maximum variation method is shown in Table 2. The cumulative variance contribution rate of the factors reached 64.648%.

**Table 2:** Load of each factor after factor rotation of formal scale for CHASQ (55 items)

Dimension name and entry content	Item source	Load
**Job satisfaction (variance contribution rate was 15.098%)**		
Do you feel that your current job has exhausted you physically and mentally?	CMI	0.496
Have you ever stopped working after going to work every day?	Qualitative interview developed	0.772
Do you feel tired after getting up for a day’s work?	CMI	0.689
Do you feel that the daily work or training intensity is too high?	Qualitative interview developed	0.640
Do you have the desire to leave work as soon as you start work?	Qualitative interview developed	0.753
Do you feel that your work is meaningless?	Qualitative interview developed	0.611
Have you been unable to lift your spirits all day?	SF-36	0.600
Do you have a strong desire to adjust your work?	Qualitative interview developed	0.637
Have you become less and less concerned about your contribution to your work?	CMI	0.625
Do you still feel tired after getting ups?	WHOQOL	0.633
Have you been dissatisfied with your financial situation?	SF-36	0.496
Have you felt tired?	SF-36	0.636
How do you feel about your overall health among your peers?	Modified from GAT	0.528
Do you suffer from loss of appetite and poor sleep during your field missions?	Qualitative interview developed	0.427
**Anxiety and depression (variance contribution rate was 11.823%)**		
Have you been worried about things?	GAD-7	0.493
Do you feel that something terrible is going to happen and you are afraid?	GAD-7	0.667
Have you any uncontrollable concerns?	GAD-7	0.674
Have you been fidgety?	GAD-7	0.698
Have you felt nervous, anxious or anxious?	GAD-7	0.566
Have you found it hard to relax?	GAD-7	0.538
Have you ever felt very defeated or disappointed yourself or your family?	PHQ-9	0.565
Have you ever been unable to work hard or lost interest?	PHQ-9	0.408
Do you feel unable to concentrate on things (such as reading books and watching TV)?	PHQ-9	0.474
Do you have any obvious slow movement or speech?	PHQ-9	0.545
Have you felt depressed, depressed or desperate?	PHQ-9	0.622
Do you think someone has plotted against you?	Modified from CMI	0.600
**Daily activities (variance contribution rate is 10.186%)**		
Has your health restricted weight-bearing activities (such as carrying/carrying things and walking)?	GAT	0.691
Has your health restricted your free activities (such as climbing stairs, jogging, etc.)?	GAT	0.749
Has your health restricted physical activity (such as bending, bending knees, squatting, etc.)?	GAT	0.698
Have you completed only part of what you wanted to do in the past year due to your health?	GAT	0.675
How healthy have you been in your daily activities (such as moving tables and chairs, cleaning, etc.)?	GAT	0.695
Have you reduced the time for work or other daily activities due to your health?	GAT	0.665
Have you restricted the types of work or activities you want to do due to your health status?	GAT	0.664
Have you found it more difficult to complete work or other things due to your health?	GAT	0.617
Has your health limited your strenuous exercise (such as rope skipping, sprint, etc.)?	GAT	0.702
Have you had any obvious pain?	GAT	0.451
**Physical function (variance contribution rate is 5.228%)**		
Has your health restricted weight-bearing activities (such as carrying/carrying things and walking)?	Qualitative interview developed	0.661
Has your health restricted your free activities (such as climbing stairs, jogging, etc.)?	Qualitative interview developed	0.755
Has your health restricted physical activity (such as bending, bending knees, squatting, etc.)?	Qualitative interview developed	0.760
**Otolaryngology system (variance contribution rate is 5.108%)**		
Do you often have a runny nose?	CMI	0.786
Have you ever sneezed hard to stop?	CMI	0.725
Do you think your nose is always blocked?	CMI	0.727
Have you had a bad cold?	CMI	0.529
**Dermal system (variance contribution rate is 6.148%)**		
Have you had a rash?	CMI	0.786
Have you ever had itchy skin?	CMI	0.749
Do you have any small bleeding spots on your skin?	CMI	0.616
Have you had any skin allergies?	CMI	0.795
**Sleep disorders (variance contribution rate was 5.635%)**		
Do you wake up early?	PSQI	0.763
Do you often dream when you sleep?	PSQI	0.694
Have you ever slept uneasily or woke up easily?	PSQI	0.747
Do you get up at night?	PSQI	0.595
**Visual system (variance contribution rate is 5.421%)**		
Have you ever had eye pain?	CMI	0.680
Have your eyes been red or inflamed?	CMI	0.727
Have you ever had blurred vision?	CMI	0.700
Have you blinked and shed tears frequently?	CMI	0.730

### Confirmatory factor analysis

The remaining 2403 sample data points were used for the CFA of the eight-factor model. The maximum likelihood estimation method was used for model fitting, and the fitting results are shown in Table 3. Except for the NFI (0.880) and the CFI (0.893; close to 0.90), the other indicators met the statistical requirements. This illustrates that the structural validity of the questionnaire was reliable and that the 8-dimensional model of the full-dimensional CHSAQ with 55 items fit well.

**Table 3:** Fitting value of confirmatory factor analysis model of CHASQ

Fitting index	Analysis results of scale data	Range
Ratio of chi square value of model fitting to degree of freedom (χ^2^/df)	4.189	≤ 5
Goodness of fit index (GFI)	0.850	≥ 0.85
Adjusted goodness of fit index (AGFI)	0.816	≥ 0.8
Specification fit index (NFI)	0.880	≥ 0.9
Nonstandard fitting index (NNFI)	0.900	≥ 0.9
Comparison fit index (CFI)	0.893	≥ 0.9
Root mean square of approximate error (RMSEA)	0.053	≤ 0.07

**Fig. 2:**
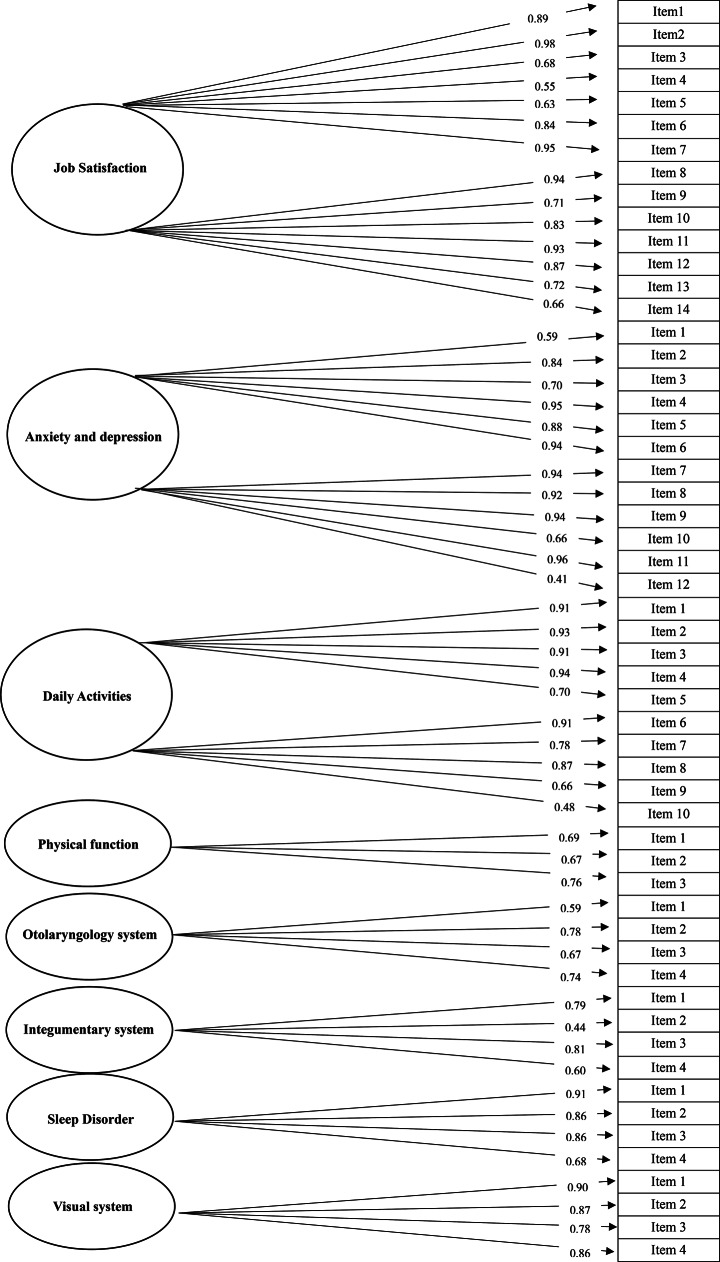
Standardized factor load model of confirmatory factor analysis. All loading scores of 55 items and 8 dimensions of CHASQ were listed in this figure

The standardized factor load model obtained from the CFA of the full-dimensional CHSAQ is shown in Fig. 2. All the items had loads greater than 0.40, and all had *P* values < 0.05.

### Content validity

The correlation coefficients between each dimension were mostly distributed in the range of 0.03–0.50, whereas the correlation coefficients between the dimension and total questionnaire scores ranged from 0.636 to 0.930 (Table 4). As shown in Table 4, the correlation coefficients between the dimension and total questionnaire scores were mostly greater than the correlation coefficient between the dimension scores. The results showed that each item could be a good representative of the whole questionnaire since each item was strongly correlated with the total score of the questionnaire. Moreover, the correlation between the dimensions was weak, which indicates that there is a certain degree of independence and discrimination between them. The content validity ratio (CVR) was 0.62, which indicated that the content validity of the questionnaire was good.

**Table 4:** Pearson correlation coefficient between the scores of each dimension and the total score of the CHASQ

Dimension name	Total score variable	Job satisfaction	Anxiety and depression	Daily activities	Physical function	Otolaryngology system	Integumentary system	Sleep disorders	Visual system
Total score variable	1.000								
Job satisfaction	0.930**	1.000							
Anxiety and depression	0.889*	0.815**	1.000						
Daily activities	0.832**	0.689**	0.693**	1.000					
Physical function	0.726**	0.662**	0.570**	0.600**	1.000				
Otolaryngology system	0.690**	0.588**	0.529**	0.509**	0.496**	1.000			
the integumentary system	0.666**	0.551**	0.534**	0.524**	0.460**	0.494**	1.000		
Sleep disorders	0.636**	0.528**	0.482**	0.443**	0.416**	0.419**	0.353**	1.000	
Visual system	0.709**	0.595**	0.551**	0.518**	0.457**	0.510**	0.468**	0.539**	1.000

*Significantly correlated at 0.05 level (bilateral)

**Significantly correlated at 0.01 level (bilateral)

### Construct validity

The results of the EFA and CFA showed that the number of dimensions envisioned in advance and that each fitting index met the statistical requirements. Furthermore, principal component analysis was conducted on each dimension of the questionnaire. Each dimension had only one factor with an eigenvalue greater than 1, and the variance contribution rate was between 58.40% and 80.25%. The loading of each item on its dimension was greater than 0.51 (Table 5), which indicated that the questionnaire had good structural validity.

**Table 5:** Principal component analysis results of each dimension of the CHASQ

dimension	Number of factors with eigenvalue > 1	Variance contribution rate (%)	Load range
Job satisfaction	1	58.40	0.67–0.85
Anxiety and depression	1	58.99	0.52–0.84
Daily activities	1	60.28	0.67–0.84
Physical function	1	80.25	0.88–0.91
Otolaryngology system	1	64.39	0.71–0.85
Integumentary system	1	70.53	0.73–0.89
Sleep disorders	1	60.62	0.66–0.87
Visual system	1	70.15	0.82–0.85

### Reliability analysis

The results of the reliability analysis of the CHSAQ are shown in Table 6. Cronbach’s α coefficient was 0.970, and for each dimension, it ranged between 0.781 and 0.944. The Guttman split-half reliability coefficient was 0.937, and the dimension coefficients ranged from 0.797 to 0.936. The test-retest reliability coefficient of 720 subjects after 2 weeks was 0.902, and the dimension coefficients ranged from 0.630 to 0.960, which indicated that the questionnaire had satisfactory test-retest reliability.

**Table 6:** Reliability coefficient of CHASQ

Dimension name	Cronbach’s α coefficient	Split half reliability coefficient	Test-retest reliability coefficient
Job satisfaction	0.944	0.936	0.896
Anxiety and depression	0.869	0.847	0.888
Daily activities	0.924	0.898	0.960
Physical function	0.876	0.797	0.885
Otolaryngology system	0.814	0.831	0.846
Integumentary system	0.856	0.811	0.915
Sleep disorders	0.781	0.800	0.881
Visual system	0.858	0.844	0.785
Scale	0.970	0.937	0.902

### Discrimination analysis results

For the CHSAQ scores, which included different demographic characteristics (service age, education level, BMI, military age, and work environment), see Table 7 for a comparison of the average scores on each dimension of the full-dimensional health assessment questionnaire. In terms of age, education level, BMI, military age and work environment, the differences in the average scores for each dimension were statistically significant (*P <* 0.05) and were reflected in different pairwise comparisons.

**Table 7:** Comparison of average scores of 8 dimensions by gender, age, education level, BMI and military age (X ± S)

Factor	Job satisfaction	Anxiety and depression	Daily activities	Physical function	Otolaryngology system	Integumentary system	Sleep disorders	Visual system
**Gender**								
Male	54.84 ± 10.68	49.26 ± 7.26	43.55 ± 6.48	11.44 ± 2.40	15.86 ± 2.92	17.48 ± 2.76	14.36 ± 3.22	16.04 ± 3.10
Female	51.22 ± 9.81^a^	47.43 ± 7.13^a^	41.47 ± 6.85^a^	10.50 ± 2.27^a^	15.57 ± 2.72^a^	16.51 ± 2.97^a^	13.63 ± 3.30^a^	15.30 ± 2.94^a^
**Age (years)**								
18 ~ 30	55.57 ± 10.47	49.72 ± 6.99	44.10 ± 6.20	11.58 ± 2.38	15.98 ± 2.93	17.60 ± 2.75	14.51 ± 3.23	16.11 ± 3.11
31 ~ 40	50.31 ± 10.75^b^	46.58 ± 8.10^b^	40.87 ± 7.15^b^	10.52 ± 2.35^b^	15.21 ± 2.80^b^	16.59 ± 2.85^b^	13.60 ± 3.16^b^	15.48 ± 2.99^b^
41~	52.13 ± 9.14^b,c^	47.82 ± 6.59^b,c^	40.28 ± 6.68^b^	10.61 ± 2.11^b^	15.62 ± 2.63	16.77 ± 2.70^b^	13.25 ± 3.07^b^	15.39 ± 2.91^b^
**Educational level**								
Junior college and below	56.73 ± 10.16	50.37 ± 6.68	44.47 ± 6.12	11.74 ± 2.39	16.22 ± 2.90	17.77 ± 2.69	14.60 ± 3.25	16.33 ± 3.10
Undergraduate course	51.53 ± 10.58^d^	47.32 ± 7.80^d^	41.95 ± 6.79^d^	10.87 ± 2.32^d^	15.30 ± 2.83^d^	16.90 ± 2.82^d^	13.83 ± 3.16^d^	15.42 ± 3.02^d^
Master and above	48.69 ± 9.69^d,e^	46.06 ± 7.19^d,e^	40.09 ± 6.72^d,e^	10.16 ± 2.06^d,e^	14.83 ± 2.66d	16.29 ± 2.91^d,e^	13.69 ± 3.12^d^	15.29 ± 2.83^d^
**BMI**								
Too light	53.57 ± 11.31	47.80 ± 7.78^f^	42.58 ± 6.83^f^	11.06 ± 2.47^f^	15.78 ± 2.89	17.42 ± 2.94^g^	14.05 ± 3.43	15.56 ± 3.33^f^
Normal	55.10 ± 10.57	49.40 ± 7.12	43.78 ± 6.37	11.56 ± 2.39	15.92 ± 2.91	17.48 ± 2.78^g^	14.41 ± 3.25^g^	16.07 ± 3.09^g^
Overweight	53.42 ± 10.72^f^	48.59 ± 7.51^f^	42.64 ± 6.81^f^	10.99 ± 2.36^f,g^	15.65 ± 2.90^f^	17.24 ± 2.82^f,g^	14.12 ± 3.19^f,g^	15.85 ± 3.05^f^
Obesity	52.59 ± 10.10^f^	48.53 ± 7.15	41.47 ± 6.80^f^	10.35 ± 2.46^f^	15.58 ± 2.91	16.61 ± 2.77^f^	13.50 ± 2.99^f^	15.30 ± 2.99^f^
**Military age (years)**								
0 ~ 10	55.36 ± 10.47	49.56 ± 7.05	43.95 ± 6.27	11.54 ± 2.39	15.96 ± 2.92	17.58 ± 2.76	14.48 ± 3.23	16.11 ± 3.10
11 ~ 20	51.08 ± 11.04^h^	47.21 ± 8.10^h^	41.36 ± 7.16^h^	10.62 ± 2.35^h^	15.28 ± 2.82^h^	16.70 ± 2.90^h^	13.62 ± 3.20^h^	15.42 ± 3.05^h^
21~	51.63 ± 9.59^h^	47.55 ± 6.83^h^	40.03 ± 6.73^h,i^	10.56 ± 2.08^h^	15.46 ± 2.81^h^	16.58 ± 2.63^h^	13.48 ± 3.00^h^	15.54 ± 2.90^h^
**Work environment**								
Normal environment	54.75 ± 10.55	49.19 ± 7.19	43.49 ± 6.46^k^	11.38 ± 2.39^k^	15.84 ± 2.90	17.41 ± 2.79	14.42 ± 3.23	16.08 ± 3.04
Extreme weather	55.28 ± 10.49	49.63 ± 6.86^k^	43.57 ± 6.49^k^	11.49 ± 2.42^k^	16.02 ± 2.92^k^	17.66 ± 2.63^k^	14.10 ± 3.20^j^	15.93 ± 3.16^k^
Confined space	52.67 ± 11.69^j^	47.97 ± 8.84	42.66 ± 6.96	11.23 ± 2.47	15.56 ± 3.01	16.90 ± 3.24^l^	13.72 ± 3.58^j^	15.32 ± 3.54^j^
Others	54.51 ± 10.65^j^	48.20 ± 7.55	42.36 ± 7.02^j^	11.01 ± 2.46	15.61 ± 2.93	17.10 ± 2.80	13.73 ± 3.09^j^	15.39 ± 3.11^j^

^a^Compared with male group, *P* < 0.05;

^b^*P* < 0.05 vs. 18–30 years old group

^c^*P* < 0.05 vs. 31–40 years old group;

^d^*P* < 0.05 vs. junior high school and below groups

^e^*P* < 0.05 vs. undergraduate group;

^f^*P* < 0.05 vs. normal weight group

^g^*P* < 0.05 vs. obesity group;

^h^*P* < 0.05 vs. 0–10 years of military service year

^i^*P* < 0.05 vs. 11–20 years of military service

^j^*P* < 0.05 vs. normal environment

^k^*P* < 0.05 vs. others

^l^*P* < 0.05 vs. extreme weather

## Discussion

The goal of this study was to describe the development and preliminary evaluation of the CHSAQ score based on health status, including physical and psychosocial strength, in China. Our preliminary data in this study showed that the CHSAQ had good test-retest reliability and construct validity. Although similar questionnaires have been developed previously, they were mainly for army personnel in developed countries such as the U.S. and western Europe. In the present study, the CHSAQ, which has 55 items and 8 dimensions, was determined to be a reliable, multidimensional assessment tool for researchers to measure personal health status among Chinese active-duty soldiers from the Chinese PLA.

It is well accepted that timely and accurate measurement of health status for military service providers and veterans is essential for improving their well-being [33, 34]. In the PLA, assessments are often confirmed using available tools such as the WHOQOL and Symptom Checklist-90 (SCL-90); nevertheless, these tools were developed for general civilians or focus on certain parts of health status measurement, such as mental health. Some questionnaires, such as the Military to Civilian Questionnaire (M2C-Q), Comprehensive Soldier & Family Fitness (CSF2), and GAT, were developed specifically for military staff in Western countries; these questionnaires include items regarding general and psychological health; employment or other meaningful activities; finances; health; life skills and preparedness; social integration; housing and physical environments; and cultural and social environments and instruments for assessing subjective health, mental health, physical health, social health and spiritual health according to the requirements of health evaluation raised by the WHO [35–37]. In the present study, we developed a comprehensive health assessment tool, specifically assessing the health of personnel in the PLA, based on the published literature and characteristics of individuals in the PLA.

To develop the CHSAQ, we conducted three rounds of surveys with a large sample size. Several statistical methods, including innovative theories, were applied to construct the questionnaire. The questionnaire was also strictly validated following the scientific process. First, several standard methods, including the discriminant analysis method, discrete trend method, correlation coefficient method, factor analysis method, and Cronbach’s α coefficient method from classic test theory (CTT), were used in accordance with previous reports [38–40]. To ensure the basic quality of each item included in the CHSAQ, two rounds of screening were conducted on all the original items, and only entries that met the requirements of four or more methods were retained. Second, in the validation stage, in addition to EFA, multiple reliability and validity testing methods were used to comprehensively validate the structure and content of the CHSAQ to further ensure the accuracy and specificity between the dimensions and the overall questionnaire.

Approximately 90% of the GAT items were adapted directly from existing measures or were slightly modified to fit the U.S. Army context. Approximately 80% of the resulting items (44/55) of the CHSAQ were adapted from well-validated and published questionnaires, such as the SF-36, the CMI and the WHOQOL instruments, which are aligned with the GAT [19]. These tools are well developed for the assessment of health quality and are used for a great number of studies. However, the questionnaires specifically developed for armies typically have different structures and applicability for targeting populations with various technical ability requirements.

For example, we modified the items on physical fitness regarding the characteristics of the Chinese PLA. Keeping soldiers at their best is crucial for successful completion of various army missions. To this end, physical fitness should be examined and monitored. In the U.S., physical fitness test data included deadlift, standing rear projection, T-push-up, 25 m-sprint/drag/carry, horizontal bar curling leg, and 2-mile-long-distance running data to comprehensively test strength, endurance, speed, coordination, sensitivity, and other physical quality indicators [41]. Nevertheless, the mission setting and requirements for the Chinese PLA are quite different from those for the U.S. army, with physical fitness test results including body type, 3-kilometre-long-distance running, horizontal bars and 30-metre × 2 running. Therefore, we developed items related to physical fitness (*n* = 3) and daily activity (*n* = 10) in the PLA [42]. Specifically, items regarding physical response, physical flexibility and explosion shots during exercise in the physical fitness dimension and working fitness and pain in the daily activities dimension were developed after quality interviews with 10 experts. These items reflected the soldiers’ physical fitness (the Cronbach’s α coefficients were 0.876 and 0.924, respectively).

In recent decades, several studies have developed self-report questionnaires for screening for mental disorders such as depression, anxiety, and insomnia [43, 44]. According to the Diagnostic and Statistical Manual of Mental Disorders-5 (DSM-5), several new self-report questionnaires, such as the PHQ-9 and GAD-7, are suggested for the rapid and easy screening of mental disorders [45]. In the CHSAQ, we included some items from these questionnaires but with slight modifications in accordance with the expert interview and two preliminary assessments of the CHSAQ.

It has been suggested that sex, area, and service years reflect outcomes in terms of epidemiological, psychological, and psychiatric studies differently [4, 39, 46, 47]. These findings indicate that these factors should be included in the development of questionnaires. In the present study, we detected significant differences in relation to personal characteristics, such as the number of years of service and education level, which also affected the results of preliminary research on the CHSAQ. This finding indicates that the health status of army personnel involves a complex interplay of age, culture and other demographic characteristics, which is consistent with the findings of Loryana L. Vie [21].

### Limitations

Notably, the CHSAQ has several limitations. First, this questionnaire does not predict psychopathological disorders, such as schizophrenia and bipolar disorders. Due to the instrument length/time constraints, we limited the time to complete the CHSAQ to no more than 20 min. Hence, items regarding numerous traits, such as psychological fitness, were deleted. According to our preliminary data, the longest time that participants confirmed that they needed to complete the CHSAQ was 15 min. However, other versions of the CHSAQ could be developed by different army units for screening for psychiatric disorders or other uses. Second, the usage frequency of the CHSAQ and the compatibility of different branches of the PLA need to be further studied. Third, this study aimed to preliminarily examine the construction and validation of the CHSAQ. Due to time limitations, we did not develop a standard, validate the patients’ health status or strictly consider the issue of confounding factors. However, whether there are confounding factors, such as the diet patterns of individuals in the PLA, still needs to be explored in further cross-sectional studies.

## Conclusions

To our knowledge, this study was the first to develop a self-report questionnaire for the assessment of the comprehensive health status of individuals in the PLA. The CHSAQ was developed to provide a theory-based tool to evaluate the health status of individuals in the PLA in terms of physical fitness, psychosocial fitness, job stratification and other dimensions. Preliminary evidence has demonstrated that the CHSAQ has good reliability, construct validity and discriminative ability.

## Data Availability

The datasets used and analysed during the current study are available from the corresponding author upon reasonable request.

## References

[1] Comtois KA, Kerbrat AH, DeCou CR, Atkins DC, Majeres JJ, Baker JC, Ries RK (2019). Effect of augmenting Standard Care for Military Personnel with brief Caring text messages for suicide Prevention: a Randomized Clinical Trial. JAMA Psychiatry.

[2] Tanofsky-Kraff M, Sbrocco T, Theim KR, Cohen LA, Mackey ER, Stice E, Henderson JL, McCreight SJ, Bryant EJ, Stephens MB (2013). Obesity and the US military family. Obes (Silver Spring).

[3] Smith TC, Zamorski M, Smith B, Riddle JR, Leardmann CA, Wells TS, Engel CC, Hoge CW, Adkins J, Blaze D (2007). The physical and mental health of a large military cohort: baseline functional health status of the millennium cohort. BMC Public Health.

[4] Kegel JL, Kazman JB, Scott JM, Deuster PA (2020). Health behaviors and Psychosocial attributes of US soldiers. J Acad Nutr Diet.

[5] Golenbock S, Kazman JB, Krauss S, Deuster PA (2017). General health status in army personnel: relations with health behaviors and psychosocial variables. Qual Life Res.

[6] Cieza A, Sabariego C, Bickenbach J, Chatterji S (2018). Rethinking disability. BMC Med.

[7] Engel GL (1977). The need for a new medical model: a challenge for biomedicine. Science.

[8] Rodway C, Ibrahim S, Westhead J, Bojanic L, Turnbull P, Appleby L, Bacon A, Dale H, Harrison K, Kapur N (2023). Suicide after leaving the UK Armed forces 1996–2018: a cohort study. PLoS Med.

[9] Wilkins KC, Lang AJ, Norman SB (2011). Synthesis of the psychometric properties of the PTSD checklist (PCL) military, civilian, and specific versions. Depress Anxiety.

[10] Kessler RC, Calabrese JR, Farley PA, Gruber MJ, Jewell MA, Katon W, Keck PE, Nierenberg AA, Sampson NA, Shear MK (2013). Composite International Diagnostic interview screening scales for DSM-IV anxiety and mood disorders. Psychol Med.

[11] Salimi Y, Taghdir M, Sepandi M, Karimi Zarchi AA (2019). The prevalence of overweight and obesity among Iranian military personnel: a systematic review and meta-analysis. BMC Public Health.

[12] Seelig AD, Jacobson IG, Donoho CJ, Trone DW, Crum-Cianflone NF, Balkin TJ (2016). Sleep and Health Resilience Metrics in a large military cohort. Sleep.

[13] Bossarte RM, Knox KL, Piegari R, Altieri J, Kemp J, Katz IR (2012). Prevalence and characteristics of suicide ideation and attempts among active military and veteran participants in a national health survey. Am J Public Health.

[14] Fikretoglu D, Brunet A, Guay S, Pedlar D (2007). Mental health treatment seeking by military members with posttraumatic stress disorder: findings on rates, characteristics, and predictors from a nationally representative Canadian military sample. Can J Psychiatry.

[15] Ware JE, Sherbourne CD (1992). The MOS 36-item short-form health survey (SF-36). I. conceptual framework and item selection. Med Care.

[16] Spitzer RL, Kroenke K, Williams JB (1999). Validation and utility of a self-report version of PRIME-MD: the PHQ primary care study. Primary care evaluation of Mental disorders. Patient Health Questionnaire JAMA.

[17] Teo BJX, Koh JSB, Jiang L, Allen JC, Yeo SJ, Howe TS (2019). Association of the 36-Item short Form Health Survey Physical Component Summary score with patient satisfaction and improvement 2 years after total knee arthroplasty. JAMA Netw Open.

[18] Peterson C, Park N, Castro CA (2011). Assessment for the U.S. Army Comprehensive Soldier Fitness program: the Global Assessment Tool. Am Psychol.

[19] Kegel JL, Kazman JB, Clifton DR, Deuster PA, de la Motte SJ (2021). Self-reported Health indicators in the US Army: longitudinal analysis from a Population Surveillance System, 2014–2018. Am J Public Health.

[20] Griffith J (2022). Cohesiveness in previously deployed Army National Guard units: implications for postdeployment behavioral health. Psychol Serv.

[21] Sullivan KS, Hawkins SA, Gilreath TD, Castro CA (2020). Preliminary psychometrics and potential Big Data uses of the U.S. Army Family Global Assessment Tool. Mil Behav Health.

[22] Jomy J, Jani P, Sheikh F, Charide R, Mah J, Couban RJ, Kligler B, Darzi AJ, White BK, Hoppe T et al. Health measurement instruments and their applicability to military veterans: a systematic review. BMJ Mil Health 2023. 10.1136/military-2022-002219 37028907

[23] Rong H, Lu L, Wang L, Liu C, Zhang L, Li F, Yi D, Lei E, Zheng C, Meng Q (2023). Investigation of health literacy status and related influencing factors in military health providers of Chinese people’s liberation Army, a cross-sectional study. BMC Public Health.

[24] Wolff HG (1946). The Cornell Indices and the Cornell Word Form; application. Ann N Y Acad Sci.

[25] Bonomi AE, Patrick DL, Bushnell DM, Martin M (2000). Validation of the United States’ version of the World Health Organization Quality of Life (WHOQOL) instrument. J Clin Epidemiol.

[26] Negeri ZF, Levis B, Sun Y, He C, Krishnan A, Wu Y, Bhandari PM, Neupane D, Brehaut E, Benedetti A (2021). Accuracy of the Patient Health Questionnaire-9 for screening to detect major depression: updated systematic review and individual participant data meta-analysis. BMJ.

[27] Gong Y, Zhou H, Zhang Y, Zhu X, Wang X, Shen B, Xian J, Ding Y (2021). Validation of the 7-item generalized anxiety disorder scale (GAD-7) as a screening tool for anxiety among pregnant Chinese women. J Affect Disord.

[28] Mollayeva T, Thurairajah P, Burton K, Mollayeva S, Shapiro CM, Colantonio A (2016). The Pittsburgh sleep quality index as a screening tool for sleep dysfunction in clinical and non-clinical samples: a systematic review and meta-analysis. Sleep Med Rev.

[29] Joseph Lee Rodgers & W (1998). Alan Nicewander: thirteen ways to look at the correlation coefficient. Am Stat.

[30] Li-tze H, Peter M, Bentler (1999). Cutoff criteria for fit indexes in covariance structure analysis: conventional criteria versus new alternatives. Struct Equation Modeling: Multidisciplinary J.

[31] Wang L, Liang C, Yu H, Zhang H, Yan X (2022). Reliability and validity evaluation of the appropriate antibiotic use self-efficacy scale for Chinese adults. BMC Public Health.

[32] Flora DB, Curran PJ, Hussong AM, Edwards MC. Incorporating measurement non-equivalence in a cross-study latent growth curve analysis. Struct Equ Modeling. 2008;15(4):676–704. 10.1080/10705510802339080 PMC2772080 19890440

[33] Jones BH, Canham-Chervak M, Canada S, Mitchener TA, Moore S (2010). Medical surveillance of injuries in the u.s. Military descriptive epidemiology and recommendations for improvement. Am J Prev Med.

[34] Moreau C, Duron S, Bedretdinova D, Bohet A, Panjo H, Bajos N, Meynard JB (2022). Mental health consequences of military sexual trauma: results from a national survey in the French military. BMC Public Health.

[35] Reivich KJ, Seligman ME, McBride S (2011). Master resilience training in the U.S. Army. Am Psychol.

[36] Gottman JM, Gottman JS, Atkins CL (2011). The Comprehensive Soldier Fitness program: family skills component. Am Psychol.

[37] Cornum R, Matthews MD, Seligman ME (2011). Comprehensive soldier fitness: building resilience in a challenging institutional context. Am Psychol.

[38] Wu C, Yan J, Wu J, Wu P, Cheng F, Du L, Du Y, Lei S, Lang H (2021). Development, reliability and validity of infectious disease specialist nurse’s core competence scale. BMC Nurs.

[39] Yuan J, Zhang Y, Xu T, Zhang H, Lu Z, Yang X, Hu M, Yu L, Yu L, Jiang X (2019). Development and Preliminary Evaluation of Chinese Preschoolers’ caregivers’ feeding Behavior Scale. J Acad Nutr Diet.

[40] Sijtsma K (2009). On the Use, the Misuse, and the very limited usefulness of Cronbach’s alpha. Psychometrika.

[41] Knapik JJ, Sharp MA, Darakjy S, Jones SB, Hauret KG, Jones BH (2006). Temporal changes in the physical fitness of US Army recruits. Sports Med.

[42] Chen Z, Du J, Hu Y, Ou K, Li H, Meng T, Zhao H, Zhou W, Li X, Shu Q (2023). Weekly cumulative extracurricular core training time predicts cadet physical performance: a descriptive epidemiological study. Heliyon.

[43] Bai W, Gui Z, Chen MY, Zhang Q, Lam MI, Si TL, Zheng WY, Liu YF, Su Z, Cheung T (2023). Global prevalence of poor sleep quality in military personnel and veterans: a systematic review and meta-analysis of epidemiological studies. Sleep Med Rev.

[44] Thomas JL, Wilk JE, Riviere LA, McGurk D, Castro CA, Hoge CW (2010). Prevalence of mental health problems and functional impairment among active component and National Guard soldiers 3 and 12 months following combat in Iraq. Arch Gen Psychiatry.

[45] Kroenke K, Spitzer RL, Williams JB (2001). The PHQ-9: validity of a brief depression severity measure. J Gen Intern Med.

[46] Smith TC, Jacobson IG, Hooper TI, Leardmann CA, Boyko EJ, Smith B, Gackstetter GD, Wells TS, Amoroso PJ, Gray GC (2011). Health impact of US military service in a large population-based military cohort: findings of the Millennium Cohort Study, 2001–2008. BMC Public Health.

[47] Valladares-Garrido MJ, Picon-Reategui CK, Zila-Velasque JP, Grados-Espinoza P, Vera-Ponce VJ, Pereira-Victorio CJ, Valladares-Garrido D, Failoc-Rojas VE (2023). Depression and anxiety in Peruvian military personnel during the pandemic context: a cross-sectional study. BMC Public Health.

